# Chemical Characterization and Biological Evaluation of New Cobalt(II) Complexes with Bioactive Ligands, 2-Picolinehydroxamic Acid and Reduced Schiff Base *N*-(2-Hydroxybenzyl)alanine, in Terms of DNA Binding and Antimicrobial Activity

**DOI:** 10.3390/ph14121254

**Published:** 2021-12-02

**Authors:** Magdalena Woźniczka, Marta Lichawska, Manas Sutradhar, Magdalena Chmiela, Weronika Gonciarz, Marek Pająk

**Affiliations:** 1 Department of Physical and Biocoordination Chemistry, Faculty of Pharmacy, Medical University of Lodz, Muszyńskiego 1, 90-151 Lodz, Poland; marta.lichawska@umed.lodz.pl (M.L.); marek.pajak@umed.lodz.pl (M.P.); 2 Centro de Química Estrutural, Instituto Superior Técnico, Universidade de Lisboa, Av. Rovisco Pais, 1049-001 Lisboa, Portugal; manas@tecnico.ulisboa.pt; 3 Department of Immunology and Infectious Biology, Institute of Microbiology, Biotechnology and Immunology, Faculty of Biology and Environmental Protection, University of Lodz, Banacha 12/16, 90-237 Lodz, Poland; magdalena.chmiela@biol.uni.lodz.pl (M.C.); weronika.gonciarz@biol.uni.lodz.pl (W.G.)

**Keywords:** cobalt(II), 2-picolinehydroxamic acid, reduced Schiff base, stabilization parameter, DNA interaction, antimicrobial activity, cytotoxicity

## Abstract

Five new heteroligand cobalt(II) complexes with 2-picolinehydroxamic acid and reduced Schiff base, *N*-(2-hydroxybenzyl)alanine, were formed in an aqueous solution over a wide pH range. The coordination properties of ligands towards the metal ion were determined using a pH-metric method, and then the speciation model was confirmed by UV–Vis studies. A stacking interaction between the Schiff base phenol ring and the 2-picolinehydroxamic acid pyridine ring was found to improve the stability of the heteroligand species, indicating more effective coordination in mixed-ligand complexes than in their respective binary systems. The antimicrobial properties of heteroligand complexes were determined against Gram-negative and Gram-positive bacteria, as well as fungal strains. The formulation demonstrated the highest bacteriostatic and bactericidal activity (3.65 mM) against two strains of Gram-negative *Helicobacter pylori* bacteria and towards *Candida albicans* and *Candida glabrata*; this is important due to the potential co-existence of these microorganisms in the gastric milieu and their role in the development of gastritis. The binary complexes in the cobalt(II)—2-picolinehydroxamic acid system and 2-picolinehydroxamic acid were not cytotoxic against L929 mouse fibroblasts, neither freshly prepared solutions or after two weeks’ storage. By comparison, the heteroligand complexes within the range 0.91–3.65 mM diminished the metabolic activity of L929 cells, which was correlated with increased damage to cell nuclei. The concentration of the heteroligand species increased over time; therefore, the complexes stored for two weeks exhibited stronger anticellular toxicity than the freshly prepared samples. The complexes formed in an aqueous solution under physiological pH effectively bound to calf thymus DNA in an intercalative manner. This DNA-binding ability may underpin the antimicrobial/antifungal activity of the heteroligand complexes and their ability to downregulate the growth of eukaryotic cells.

## 1. Introduction

The development of resistance to antibiotics and other agents, such as anticancer, antifungal and antiviral drugs, is now a major problem in clinical practice because it reduces the effectiveness of treatment, causing increased morbidity and mortality [1,2,3,4,5,6]. Two microorganisms, *H. pylori* and *Candida*, have been found to demonstrate synergy in the development of gastric diseases [7]. When looking for new drugs, a range of pharmacologically promising compounds should be evaluated, and this evaluation should include their chemical and biological properties. Heteroligand structures play an important role in biological processes [8,9,10,11], and model systems containing aromatic amines as the primary ligand and amino acid derivatives as the secondary ligand could be used to better understand the transport of metal ions and active substances through membranes and biochemical reactions catalyzed by metalloenzymes [12]. The aromatic properties improve the lipophilicity of the complexes [13], thus increasing the permeability through the cell wall [14]. However, to confirm the structure and stability of a particular heteroligand species, it is necessary to determine the interaction between two ligands bound to the same metal ion.

The effects of transition metal complexes on DNA and their biological applications have aroused great interest in recent years. In particular, cisplatin is the basis for many DNA-disrupting compounds [15]. Given the important role played by DNA, drugs that are able to directly interact with DNA can be highly effective at killing cells [16]. Unfortunately, their products can have severe side effects. To overcome these limitations, other classes of metallic drugs, which show interactions with DNA but with lower toxicity are under evaluation [17]. Moreover, bioorganometallic compounds can be oriented towards structures other than DNA, for example enzymes [18,19]. In complex systems, ligands containing electron deficiencies on the pyridine and phenol rings more readily bind to the DNA helix and as such are attractive compounds in drug design [20,21].

**Figure 1:**
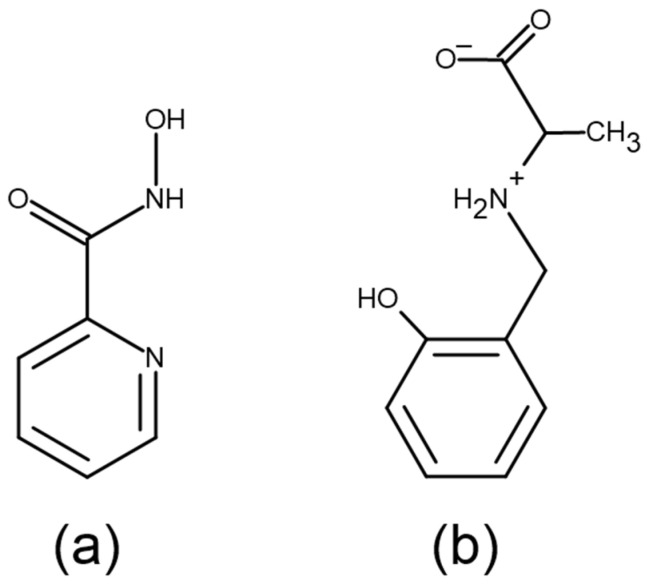
Ligand structures (**a**) 2-picolinehydroxamic acid, PicHA (LH) (**b**) *N*-(2-hydroxybenzyl)alanine, AlaSal (L’H_2_).

The present study attempts to characterize the biological activities of cobalt(II) systems. Such transition metal complexes provide possibilities of creating new therapeutic products unavailable to organic compounds due to their varied coordination numbers, geometries, redox states and thermodynamic–kinetic characteristics. Cobalt(II), as the central ion of vitamin B_12_ and an enzyme component, is essential for the normal functioning of living organisms [22]. Due to the emergence of drug resistance related to the platinum chemotherapeutic agents, drugs based on cobalt complexes have shown encouraging results, also supported by low expenditure for the design of new species [23,24]. Chelation affects a distinct change in the biological properties of the ligands and the metal moiety; their presence can cause a range of diseases, including cancer; however, they can also be used to treat them [1]. To obtain biologically active metal complexes, it was advisable to use ligands such as 2-picolinehydroxamic acid (PicHA) and *N*-(2-hydroxybenzyl)alanine (AlaSal), Figure 1, which are known to demonstrate antibacterial and antifungal properties [25,26].

The coordination chemistry of Schiff base ligands has been the subject of great interest due to their strong metal binding ability and stabilize them in various oxidation states [27,28,29]. The Schiff base is derived from an amino acid and a carbonyl compound (e.g., salicylaldehyde). The reduction of its C=N bond to a single amine bond confers greater conformational flexibility to the backbone, allowing the compound to form complexes with more stable molecules [26]. Moreover, aromatic Schiff bases are a more stable class of compounds than those with alkyl substituents, which are relatively unstable and readily polymerizable [30]. Schiff basic complexes are active in photosynthesis and oxygen transport in the respiratory systems. In addition, they are used for the liquid membrane transport of metal cations, liquid-liquid extraction and as catalysts [31]. Schiff base complexes possess antifungal [32] and antibacterial [1,33] properties and can bind to DNA [34], as well as antitumor activity, influenced by the presence of an electron withdrawing group in the benzene ring [20,35]. Heterocyclic compounds containing pyridine and its related compounds, such as PicHA, offer promise as biological ligands due to the presence of a ring nitrogen with a localized pair of electrons. Their effectiveness against bacterial and fungal pathogens was enhanced when coordinated with transition metal ions [20]. Moreover, heteroligand Co(II) complexes of salicylic acid with pyridine showed a pronounced antimicrobial and fungicidal effect [36].

The present study compares the antibacterial and antifungal activity of new Co(II)–PicHA–AlaSal complexes with those of PicHA alone and Co(II)–PicHA complexes [25], and examines the effect of storage for two weeks. It also determines the range of cytotoxic activity of the studied complexes and ligand forms towards eukaryotic cells.

## 2. Results and Discussion

### 2.1. Heteroligand Complex Formation Equilibria

**Figure 2:**
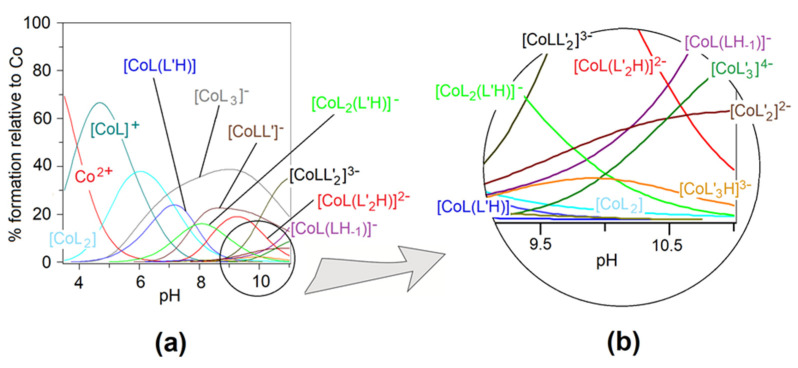
(**a**) Distribution diagram of species as a function of pH relative to Co(II), for the complexes formed in the Co(II)–PicHA–AlaSal system at PicHA:AlaSal:Co(II) molar ratio 2:2:1, *C*_Co(II)_ = 5.0 × 10^−3^ M, (**b**) extended part of species distribution curves.

Heteroligand complexes are formed as a result of the cobalt(II) ion coordination with deprotonated functional groups of PicHA and AlaSal. Dissociation of both ligands begins at pH below 2 [25,26]. However, only the binary complexes with PicHA are formed in the {N_pyr_,N^−^} chelation mode in these conditions (Figure 2). In turn, participation in the complexation of the AlaSal carboxyl group is possible only in the presence of an amine nitrogen [37]. Thus, the formation of the complex with the participation of AlaSal is possible at pH values above 4 [26].

**Figure 3:**
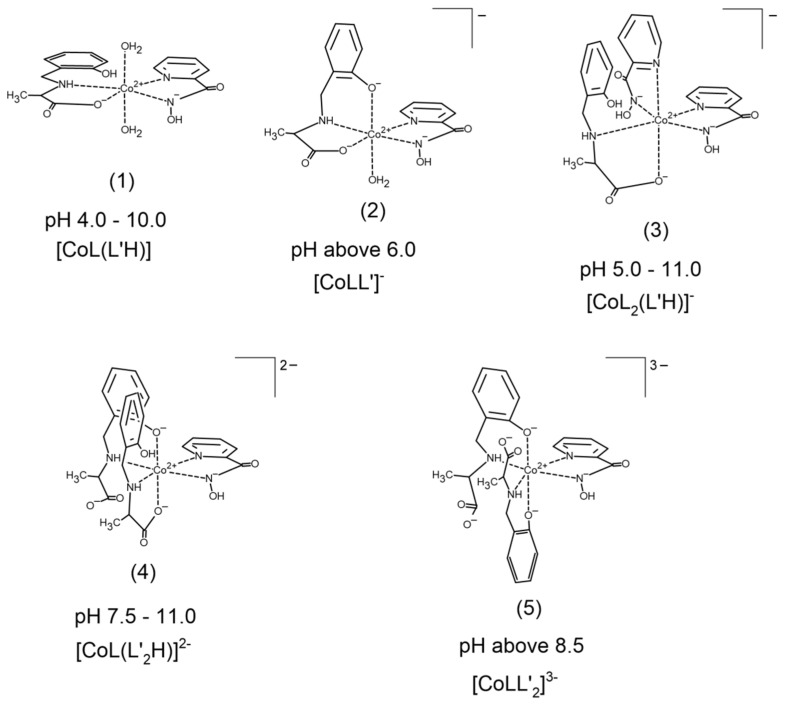
Proposal for pH-dependent coordination of complexes formed in the Co(II)–PicHA–AlaSal system.

Five heteroligand complexes (Figure 3) were confirmed by potentiometry; the overall stability constants together with the related constants are presented in Table 1. As it can be seen in the speciation diagram (Figure 2), the first heteroligand [CoL(L’H)] complex (**1**) occurs at pH 4 (Figure 3). The AlaSal with protonated phenolic group most likely coordinates the metal via carboxyl and amine atoms in place of equatorial water molecules of the binary PicHA complex, [CoL]^+^. This chelation mode has been previously described for an analogous binary X-ray crystal form of hydroxybenzyl derivative [38]. At higher pH values, the equilibrium set comprises a complex with a completely deprotonated AlaSal molecule [CoLL’]^−^ (**2**) (Figure 3). The deprotonation step of the **1** complex forming **2** occurs according to the equation:

[CoL(L’H)] = [CoLL’]^−^ + H^+^ (1)

hence the deprotonation constant $pK_{\mathrm{CoLL}\prime }^{\mathrm{CoLL}\prime H}=\log_{10}\beta_{\mathrm{CoLL}\prime H}-\log_{10}\beta_{\mathrm{CoLL}\prime }=21.40-13.56=7.84$ reaches a lower value than the deprotonation constant for the phenolic group of free AlaSal (p*K*_aOH_ = 10.73) [26]. Following metal promoted deprotonation, the **2** complex could be formed at a pH slightly above 6 (Figure 2). The phenolic oxygen of the AlaSal molecule is most likely bonded in the axial position, while the amine nitrogen and carboxyl oxygen remain in equatorial sites.

The stabilization constant ($\Delta \log_{10}\beta$) [39] indicates the stability of heteroligand complexes compared to the corresponding binary systems. $\Delta \log_{10}\beta$ was calculated for **2** complex using the overall stability constants of the parent PicHA and AlaSal bi-ligand species, being 11.61 [25] and 13.35 [26], respectively, based on the following equations:

(2) $$\log_{10}\;\beta_{stat}=\log_{10}2+0.5\log_{10}\beta_{{\mathrm{CoL}}_{2}}+0.5\log_{10}\beta_{{\mathrm{CoL}\prime }_{2}}=\log_{10}2+0.5\cdot 11.61+0.5\cdot 13.35=12.78$$

(3) $$\mathrm{and}\;\Delta \log_{10}\beta_{\mathrm{CoLL}\prime }=\log_{10}\beta_{\mathrm{CoLL}\prime }-\beta_{stat}=13.56-12.78=0.77$$

The disproportionation constant$\;\log_{10}X$ [40], like $\Delta \log_{10}\beta$, also defines the stability of the heteroligand complexes relative to the binary complexes with two ligand molecules:

(4) $$\log_{10}X_{\mathrm{CoLL}\prime }=2\log_{10}\beta_{\mathrm{CoLL}\prime }-\log_{10}\beta_{{\mathrm{CoL}}_{2}}-\log_{10}\beta_{{\mathrm{CoL}\prime }_{2}}=2\cdot 13.56-11.61-13.35=2.16_{\;}$$

A higher $\Delta \log_{10}\beta_{\mathrm{CoLL}\prime }$ value was observed compared to previous studies [41], and the $\;\log_{10}X_{\mathrm{CoLL}\prime }$ value was more positive [39]; these indicate that the heteroligand complex has a very high degree of stability. In the case of the binary Co(II)–AlaSal system, the complexation of two tridentate molecules is less favoured than for the heteroligand species, where the ligands occupy five coordination sites in the octahedral mode.

In turn, $\;\Delta \log_{10}K$ [39] is used to compare the stabilization of the heteroligand complexes with their parent systems containing one ligand molecule; it is calculated based on the difference between the stability constants of the **2** (Table 1) and two binary [CoL]^+^ ($\log_{10}\beta_{\mathrm{CoL}}$ = 6.44) [25] and [CoL’]^+^ ($\log_{10}\beta_{\mathrm{CoL}\prime }$ = 7.98) [26] complexes:

(5) $$\;\Delta \log_{10}K_{\mathrm{CoLL}\prime }=\log_{10}\beta_{\mathrm{CoLL}\prime }-\log_{10}\beta_{\mathrm{CoL}}-\log_{10}\beta_{\mathrm{CoL}\prime }=13.56-6.44-7.98=-0.86$$

Complex **2** demonstrates lower stability than the binary complexes with one PicHA molecule or one AlaSal molecule, as indicated by the negative value of $\;\Delta \log_{10}K_{\mathrm{CoLL}\prime }$. This is due to the binary complexes demonstrating a greater availability of coordination positions for bonding the first ligand rather than coordinating the second ligand to form a heteroligand structure [39]. An exception is the positive value of$\;\Delta \log_{10}K$ for the heteroligand complex **1** with a protonated phenolic group of AlaSal, where $\log_{10}\beta_{\mathrm{CoL}\prime H}$ = 13.64 [26]:

(6) $$\;\Delta \log_{10}K_{\mathrm{CoL}(L\prime H)}=\log_{10}\beta_{\mathrm{CoL}(L\prime H)}-\log_{10}\beta_{\mathrm{CoL}}-\log_{10}\beta_{\mathrm{CoL}\prime H}=21.40-6.44-13.64=1.32$$

The obtained value is more positive than those given for other heteroligand systems [9,40], most likely due to the presence of a phenol ring affecting the stability of the heteroligand complex. It appears that the stacking interaction occurring between the AlaSal aromatic ring, which is flexible, and the PicHA pyridine ring, which is rigidly attached to the metal ion in an equatorial position, results in the heteroligand complex demonstrating greater stability than the binary complex [CoL’H]^+^ with one protonated molecule [41].

In the pH range of the **1** and **2** formation, the [CoL_2_(L’H)]^−^ complex (**3**) was also observed (Figure 2). The **3** complex (Figure 3) probably arose due to the attachment of the protonated AlaSal molecule to the previously formed [CoL_2_] complex with PicHA. In order to define the coordination mode in the complex, the$\;\Delta \log_{10}K$ was calculated according to the equation:

(7) $$\;\Delta \log_{10}K_{{\mathrm{CoL}}_{2}\left(L\prime H\right)}=\log_{10}\beta_{{\mathrm{CoL}}_{2}\left(L\prime H\right)}-\log_{10}\beta_{{\mathrm{CoL}}_{2}}-\log_{10}\beta_{\mathrm{CoL}\prime H}=25.61-11.61-13.64=0.36$$

The parameter is more positive than the predicted values for octahedral geometries [9]. This indicates that the addition of protonated AlaSal as the third molecule allows higher stabilization of the heteroligand complex **3** than its primary analogs. Therefore, as with **1**, there should also be a stacking interaction between AlaSal and PicHA rings (Figure 3). However, coordination of all positions by the AlaSal and PicHA in the heteroligand complex results in an increase of mutual electronic effects and the complex **3** than **1**. The equatorial positions of the **3** complex are most likely occupied by four nitrogen atoms, three of which are from PicHA molecules and one from AlaSal. In turn, the pyridyl nitrogen and carboxyl oxygen probably bind to the central ion at axial sites, thus confirming the interaction between the rings.

An alkaline medium promotes the formation of two successive heteroligand complexes (Figure 2): [CoL(L’_2_H)]^2−^ (**4**) (Figure 3) and after phenolic group deprotonation [CoLL’_2_]^3−^ (**5**) (Figure 3), where the deprotonation constant $pK_{{\mathrm{CoLL}\prime }_{2}}^{{\mathrm{CoL}(L\prime }_{2}H)}=9.88$. Coordination of the second AlaSal molecule undoubtedly reduced the stability of both species in relation to binary complexes, as confirmed by the negative values of the parameters:

(8) $$\Delta \log_{10}K_{{\mathrm{CoL}(L\prime }_{2}H)}=\log_{10}\beta_{{\mathrm{CoL}(L\prime }_{2}H)}-\log_{10}\beta_{\mathrm{CoL}}-\log_{10}\beta_{{\mathrm{CoL}\prime }_{2}H}=26.75-6.44-21.78=-1.47$$

(9) $$\Delta \log_{10}K_{{\mathrm{CoLL}\prime }_{2}}=\log_{10}\beta_{{\mathrm{CoLL}\prime }_{2}}-\log_{10}\beta_{\mathrm{CoL}}-\log_{10}\beta_{{\mathrm{CoL}\prime }_{2}}=16.87-6.44-13.35=-2.92$$

The presence of protonated O_phenolic_ atom and the associated flexibility of the AlaSal ring probably allow the stacking interaction between phenol rings (Figure 3). However, the AlaSal carboxyl oxygen is negatively charged and fewer coordination sites are available than the donor groups; this could be the reason for the destabilization of **4** complex, as does the electrostatic repulsion between negatively charged carboxyl oxygens in **5** (Figure 3) [42,43,44].

### 2.2. UV–Vis Spectra

Starting from a pH above 2.0, the UV-Vis absorption spectra for the Co(II)–PicHA–AlaSal system indicates a blue shift of the *d-d* bands relative to the Co(H_2_O)_6_^2+^ aqua-ion, 515 nm (molar absorption coefficient *ε* = 4.6) [45] (Figure S1a,b). This suggests the formation of binary octahedral Co(II)–PicHA complexes, as confirmed by previous studies [25]. Reaching a pH of 3.87, as in the potentiometric titration (Figure 2), allowed the formation of an octahedral heteroligand **1** complex, confirmed both by a color change from transparent to light brown and HypSpec deconvolution (Figure S1c). The high stabilization of the complex, caused by the interaction between ligand rings and enhanced by the presence of the donor –OH group, increased the charge transfer (CT) absorption band [46]. Further alkalization resulted in the formation of three subsequent heteroligand complexes: **3**, **4** and **5** (Figure S1c), for which the CT absorption bands appear to be mixed with the *d-d* transition bands [47]. The **2** complex has not been identified, probably due to the simultaneous occurrence of other species in a very similar pH range (Figure 2).

### 2.3. DNA-Binding Studies of Co(II) Complexes

**Figure 4:**
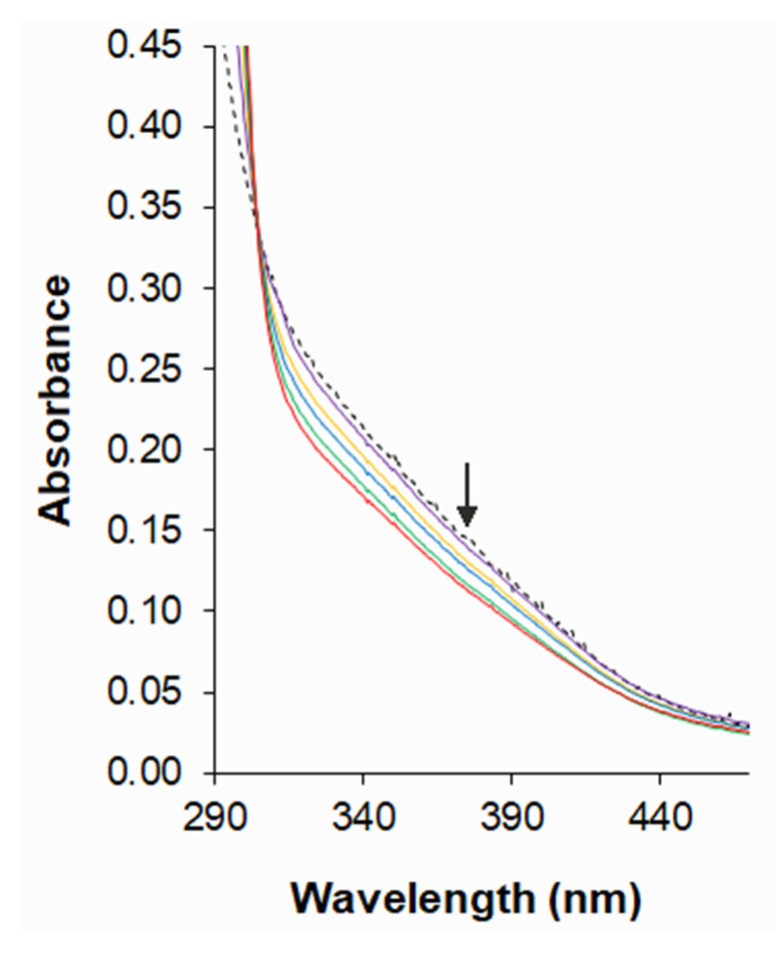
Electronic absorption spectra of species in Co(II)–PicHA–AlaSal system (*C*_Co(II)_ = 6.5 × 10^−5^ M, at PicHA:AlaSal:Co(II) molar ratio 2:2:1) in 5 mM Tris-HCl/NaCl buffer with increasing concentration of CT-DNA (2.50 × 10^−4^–1.88 × 10^−3^ M) at pH 7.2. Dashed line corresponds to the Co(II)–PicHA–AlaSal complexes in the absence of CT-DNA. Arrow indicates the absorbance change upon increasing CT-DNA concentration.

The ability to bind heteroligand cobalt(II) complexes with DNA was determined by the electron absorption method. The spectra indicated that the heteroligand system was stable over the 24 h that the assays were performed (Figure S2). At about 304–440 nm, a decrease of absorbance was observed for subsequent Co(II)–PicHA–AlaSal samples containing increasing concentrations of DNA sodium salt from calf thymus (CT-DNA) (Figure 4). The changes in absorbance suggest that the heteroligand complexes bind to DNA by intercalation, due to the strong stacking interaction between planar aromatic chromophores of the ligands and the DNA base pairs. Such interaction has already been observed for the PicHA binary complexes [25] as well as for other Co(II) structures containing pyridyl groups [48] and reduced Schiff base ligands [49]. The stacking interaction between the π*-orbital of the intercalated ligand and the π-orbital of the DNA base pairs leads to a decrease in the energy levels of the complex, causing the π-π* transitions to decrease, resulting in hypochromism in the absorption spectra [50]. The electron-withdrawing effect of the ligand rings can increase the affinity of the complexes to DNA [51]. Moreover, a strongly flattened PicHA conformation favors the formation of the intercalative binding mode [52,53].

### 2.4. Biological Activity

The minimal inhibitory concentration (MIC) for bacterial and fungal strains and minimal bactericidal concentration (MBC) or minimal fungicidal concentration (MFC) values for the Co(II)–PicHA–AlaSal complexes, showing antibacterial and antifungal activity, respectively, are summarized in Table 2, along with the MIC/MBC/MFC for standard antibiotics (gentamicin, amphotericin B and amoxicilicin). Due to the time-dependent structural change of the binary Co(II)–AlaSal complexes described previously [26] and the possibility of comparing the antimicrobial activity of these species with heteroligand complexes, the biological activity was studied for two types of solutions: one prepared directly and another that had been stored for two weeks at room temperature 25 °C and protected from light. However, no differences in the MIC and MBC/MFC values were observed for the two groups of solutions. The antibacterial and antifungal activity for Co(II), PicHA and AlaSal as well as their cobalt(II) binary systems have been tested in previous studies [25,26]. In order to consider the differences in molecular weight, the MIC/MBC/MFC values were expressed in mM, which allowed the antimicrobial activity of the heteroligand complexes to be compared with the reference data for the ligands and parent complexes.

The Co(II)–PicHA–AlaSal complexes exhibited the highest bacteriostatic and bactericidal activity against two *H. pylori* strains, for which MIC and MBC were 3.65 mM; at this value, the complexes also inhibited the growth of *C. albicans* and *C. glabtrata* and showed fungicidal activity. Earlier studies based on cobalt(II) heteroligand complexes in the solid state also found these systems to have high activity against *C. albicans* [54]. Clinical data support that *H. pylori* and *Candida* demonstrate the synergy in the development of gastric ulcers [7]. The binary Co(II)–AlaSal complexes were equally active against both strains, while AlaSal alone showed lower activity [26]. This is most likely due to the formation of chelates with greater lipophilicity and permeability through the bacterial cell membrane [55]. In turn, PicHA alone, as an inhibitor of bacterial urease [56], exhibits similar MIC values against *H. pylori* as the heteroligand complexes, and has higher activity against these strains than the binary Co(II)–PicHA complexes [25]. The Co(II)–PicHA–AlaSal complexes demonstrated the lowest MIC and MCB values (7.30 mM) against *P. aeruginosa*, *E. coli* and *E. faecalis* (Table 2). They also displayed MIC values of 3.65 mM and MBC values of 7.30 mM against *S. auresus*, *S. epidermidis* as well as *C. parapsilosis*. Most likely, the aromatic nature of the ligands and the presence of electron-donating amine and hydroxyl groups can influence the antimicrobial activity of the complexes [13,14,57,58]. The tested bacteria and fungi had varying sensitivity to the antimicrobial effects of the Co(II)–PicHA–AlaSal complexes, probably due to their different cell structures. The tested formulations showed significantly different MIC/MBC/MFC values to classic antibiotics, probably due to the fact that the values for the aqueous Co(II)–PicHA–AlaSal system were determined for the mixture of complexes as well as two ligand forms: [LH] and [L’H_2_] present in this solution at pH 7.2 (cf. Figure 2, Figures S3 and S4). [LH] occurs in a small percentage in the system; however, despite having the dominant share, [L’H_2_] shows a lower antimicrobial activity than those of the complexes (cf. Table 2, [26]).

**Figure 5:**
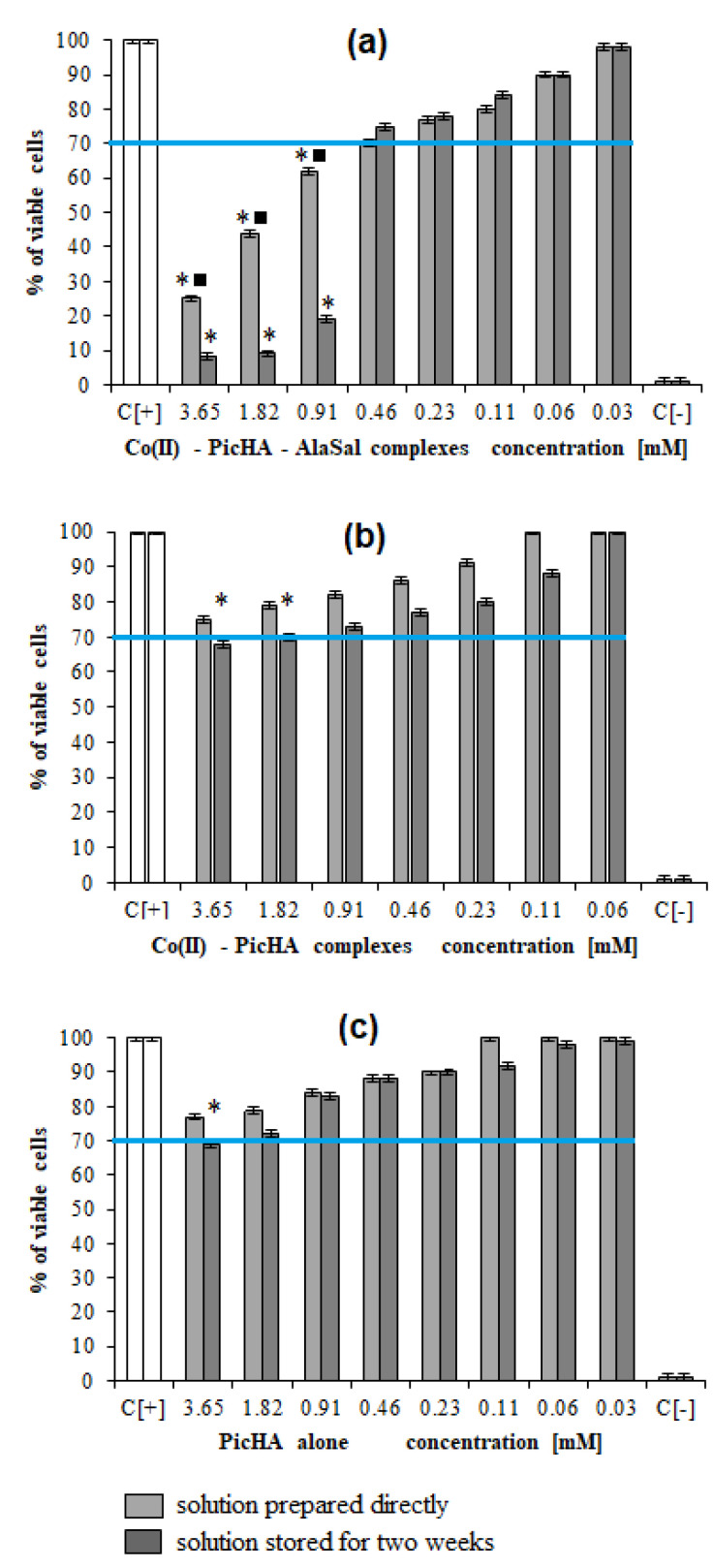
Cytotoxic effect of the studied complex and ligand forms: (**a**) Co(II)–PicHA–AlaSal, (**b**) Co(II)–PicHA and (**c**) PicHA towards mouse fibroblasts L929. The cytotoxicity was assessed by 3-(4,5-dimethylthiazol-2-yl)-2,5-diphenyltetrazolium bromide (MTT) reduction assay. The cell viability was calculated for four experiments including three repeats for each compound. Complete RMPI-1640 medium (cRPMI) was used as a positive control (C+) of cell viability (100% viable cells) and 0.03% H_2_O_2_ as a negative control (C−) of cell viability (100% dead inactive cells). Statistical significance: * ■ *p* < 0.05; * untreated cells vs. cells treated with tested solution (solution prepared directly or solution stored for two weeks); ■ solution prepared directly vs. solution stored. The blue line indicates the minimal percentage of viable cells (70%) required to confirm the compound as noncytotoxic at the in vitro level. Data are presented as mean values ± SD.

The complexes in the Co(II)–PicHA–AlaSal system significantly diminished the ability of L929 cells to reduce 3-(4,5-dimethylthiazol-2-yl)-2,5-diphenyltetrazolium bromide (MTT) (30–92% of dead cells) within the range 0.91–3.65 mM (Figure 5a). The ensuing heteroligand complexes enhance the possibility of stacking and intercalation through a series of noncovalent interactions with DNA, which may lead to an enhanced cytotoxic effect [23]. The complexes stored for two weeks have a stronger inhibitory effect within the concentration range of 0.91–3.65 mM than the freshly prepared mixtures; this may be due to the concentration of the heteroligand complexes increasing over time, and would be in line with the increase of absorbance of the UV–Vis spectra for the Co(II)–PicHA–AlaSal system at pH about 7 (Figure S2). A combination of the high concentration of the AlaSal zwitter-ionic form [L’H_2_] (Figure S4a) and the two-week delay needed for equilibration of the system most likely allowed the formation of [L’H]^−^ and its coordination with binary PicHA complexes, resulting in the production of heteroligand species. In addition, the possibility of deprotonation of PicHA as the [LH] form to the complex-forming ion [L]^−^ enables the formation of Co(II)–PicHA complexes, and then their coordination with AlaSal. The Co(II)–PicHA structures, and the AlaSal and PicHA ligands, show a weaker cytotoxic effect towards mouse fibroblasts L929 than the heteroligand complexes (Figure 5b,c, [26]). Therefore, the increasing inhibition of cell growth after two weeks of storage may be attributed to decreasing share of ligands and binary complexes in the solution and the increase of the concentration of more cytotoxic heteroligand structures (Figure 5a).

**Figure 6:**
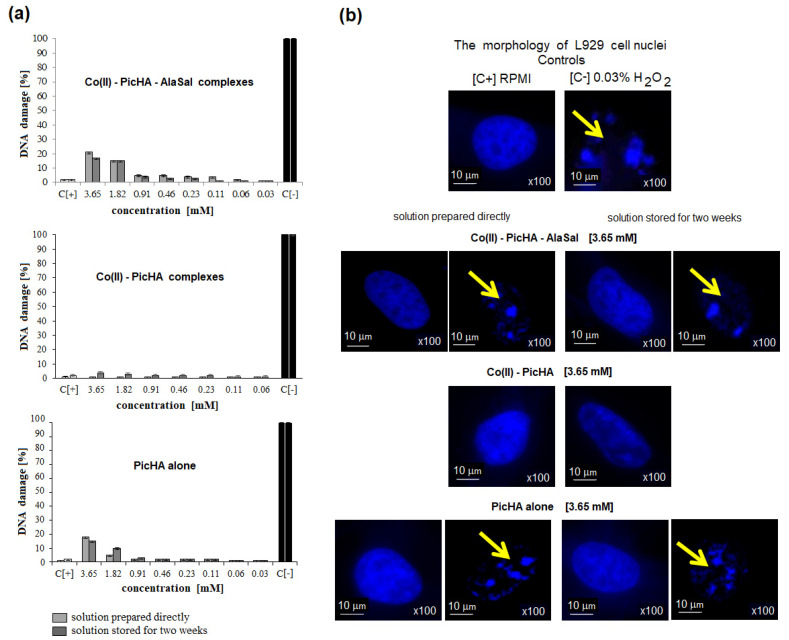
Percentage of L929 cells with vesicular cell nuclei (**a**) and representative microscopic images of L929 cells with signs of cell nuclei damage (**b**). The cells were stimulated for 24 h with complex or ligand forms. Cell cultures in complete RMPI-1640 medium (cRPMI) were used as positive controls (C+): cells with no signs of cell nuclei damage; cells treated with 0.03% H_2_O_2_ were used as negative controls (C−): cells with DNA damage. The morphology of cell nuclei was assessed by 4′,6-diamidino-2-phenylindole (DAPI) staining. Samples were viewed under a fluorescent microscope at 100× magnification (Axio Scope A1, Zeiss). Data are presented as mean values ± SD. Yellow arrows indicate cells with signs of cell nuclei degradation.

In cell cultures exposed to 1.82–3.65 mM Co(II)–PicHA–AlaSal complexes or PicHA alone, freshly prepared or stored, approximately 20% of the cells showed a tendency to cell nuclei vesication (Figure 6a) compared to unexposed controls; however, this difference was not statistically significant. In the presence of Co(II)–PicHA, the cell nuclei remained almost unchanged in the entire cell population (Figure 6a).

The complexes Co(II)–PicHA–AlaSal at a concentration 3.65 mM induced the DNA damage in about 20% of cells. By comparison, PicHA showed at this concentration (3.65 mM) nearly equal effect (Figure 6). However, PicHA (3.65 mM) alone can be considered as non- or low cytotoxic (viable cells about 70%), whereas the Co(II)–PicHA–AlaSal complexes (3.65 mM) show strong cytotoxic effect (viable cells less than 30%) (Figure 5), although they induce the same extent of DNA damage as PicHA alone. It could be due to different intracellular targets for PicHA and Co(II)–PicHA–AlaSal complexes. Recently Law et al. [59], have identified a panel of cobalt complexes, which were able to inhibit growth of multidrug-resistant cancer cells due to induction of autophagy (type II programmed cell death), cell cycle arrest (increased population of cells in S-phase), inhibition of cell invasion (downregulation of expression of metastasis-related metalloproteinase-9 and intercellular adhesion molecule-1), and inactivation of P-glycoprotein, which in taxol-resistant cancer cells is associated with the upregulation of cytosolic drug efflux.

## 3. Materials and Methods

### 3.1. Materials

The procedures for the synthesis of the 2-picolinehydroxamic acid (PicHA) and *N*-(2-hydroxybenzyl)alanine (AlaSal) have already been reported in [60,61], respectively. Cobalt(II) nitrate hexahydrate and cobalt(II) perchlorate hexahydrate from Sigma-Aldrich ( Saint Louis, MO, USA) were titrated as described in [25]. Alkali solutions (0.1 M and 1.0 M NaOH, carbonate-free) were purchased from J.T. Baker (Avantor, Radnor, PA, USA). The solutions of the HNO_3_ and HClO_4_ from Sigma-Aldrich, Saint Louis, MO, USA, were determined by the Gran method [62]. Potassium nitrate(V) (J.T. Baker, Avantor, Radnor, PA, USA) and sodium perchlorate monohydrate (Sigma-Aldrich, Saint Louis, MO, USA) were used to adjust the ionic medium. DNA sodium salt from calf thymus (CT-DNA) and Tris-HCl from Sigma-Aldrich (Saint Louis, MO, USA), sodium chloride (Chempur, Piekary Śląskie, Poland) and argon of high purity (Linde, Dublin, Ireland) were used.

Mueller–Hinton liquid medium (Biocorp, Issoire, France), Brucella medium (Biocorp, Issoire, France), supplemented with 10% fetal bovine serum (FBS) (Sigma-Aldrich, Saint Louis, MO, USA), RPMI-1640 with or without phenol red, amphotericin B, gentamicin, amoxicillin, penicillin, streptomycin, trypsin, 3-(4,5-dimethylthiazol-2-yl)-2,5-diphenyltetrazolium bromide (MTT), 4′,6-diamidino-2-phenylindole (DAPI) (all from Sigma-Aldrich, Saint Louis, MO, USA) were applied in the biological studies.

### 3.2. pH-Metric Titrations

Potentiometric titrations have been determined by using an automatic titrator system Titrando 905 (Metrohm, Herisau, Appenzell Ausserrhoden, Switzerland). A combined glass electrode LL Biotrode (Metrohm, Herisau, Appenzell Ausserrhoden, Switzerland) was calibrated daily on the hydrogen ion concentration [63]. The measurements were carried out in a thermostatted vessel at constant temperature 25.0 ± 0.1 °C and ionic strength 0.1 M (KNO_3_). Pure argon was passed over the solution surface [25].

The formation constants of heteroligand complexes in the aqueous Co(II)–PicHA–AlaSal system were carried out at metal concentrations within the range of 5.0–10.0 × 10^−3^ M. The system was tested at PicHA:AlaSal:Co(II) molar ratios of 1:1:1, 2:2:1 and 3:2:1 in a pH range of approximately 3.5–11.0.

The fitting procedure using Hyperquad 2013 software allowed the calculation of the concentration formation constants according to the formula: *β_mll’h_* = [M*_m_*L*_l_*L’*_l’_*H*_h_*]/[M]*^m^*[L]*^l^*[L’]*^l’^*[H]*^h^* [64]. PicHA and AlaSal dissociation constants, the overall stability constants of binary complexes in the Co(II)–PicHA and Co(II)–AlaSal systems, as well as the hydrolysis constants of cobalt(II) were used in the Hyperquad model and taken from own data [25,26]. All formation constants were determined under the same conditions as the heteroligand species. The ionic product of water p*K*_w_, included in the equilibrium model was 13.77 [65]. The speciation diagrams were simulated in HySS 2009 [66].

### 3.3. Spectrophotometric Measurements

UV–Vis experiments were carried out by means of a Cary 50 Bio spectrophotometer with 5 mm fiber-optic device (Varian Pty. Ltd., Mulgrave, Australia), corresponding to a 1 cm path length. The fiber-optic probe was dipped directly into the thermostatted titration vessel. A fiber optic probe, immersed in the titration vessel of a Titrando 905 automatic titration system (Metrohm, Herisau, Appenzell Ausserrhoden, Switzerland), enabled spectrophotometric measurements with simultaneous control of pH values. Since the nitrate ions show a significant absorption near 300 nm (*ε* ≈ 8), all the UV measurements were carried out in perchlorate medium [67], using a combined polymer microelectrode InLab Semi-Micro (Mettler-Toledo, Columbus, OH, USA, Stany Zjednoczone). Therefore pH and ionic strength (*I* = 1.0 M) were adjusted by HClO_4_ and NaClO_4_, respectively. Perchlorate salts as potentially explosive were used in small quantities and handled with care. Prior to use, the electrode was standardized with buffers at pH 4.00 and 7.00. All experiments were carried out in an aqueous solution at 25.0 ± 0.1 °C, with argon flowing over the surface, and titrated with carbonate-free NaOH. During titration, the pH and EMF were monitored by the Titrando 905 titrator (Metrohm, Herisau, Appenzell Ausserrhoden, Switzerland). The spectra were recorded with a slow scan (300 nm·min^−1^) [26].

The Co(II) complexation with PicHA and AlaSal were performed at PicHA:AlaSal:Co(II) molar ratio 2:2:1, at metal concentration 1 × 10^−3^ M within a pH range of approximately 2.0–11.5. The studies were carried out within the wavelength range of 200–700 nm. The molar absorption coefficients of the individual species were calculated by the HypSpec program [64].

### 3.4. DNA-Binding Experiment

The binding of the Co(II)–PicHA–AlaSal complexes to CT-DNA was evaluated by electronic absorption using a Cary 50 Bio spectrophotometer (Varian Pty. Ltd., Mulgrave, Australia) with coupled via a fiber-optic device with the Titrando 905 automatic titrator (Metrohm, Herisau, Appenzell Ausserrhoden, Switzerland) [25]. A constant temperature of 25.0 ± 0.1 °C was maintained. Solutions for both the Co(II)–ligand system and the CT-DNA were prepared in an aqueous 5 mM Tris-HCl/NaCl buffer adjusted to a pH of about 7.2. The purity of the CT-DNA solution, sufficiently free of protein, was confirmed by the 260:280 nm UV absorbance ratio remaining within the range of 1.81–1.85 [68].

Six solution samples of the Co(II)–PicHA–AlaSal system were prepared with a PicHA-to-AlaSal-Co(II) molar ratio of 2:2:1 and a total metal concentration of 6.5 × 10^−5^ M in each sample. Five samples additionally contained CT-DNA solutions at increasing concentrations, while one sample was without DNA. The concentration of CT-DNA in solution determined based on the known molar absorption coefficient of 6650 M^−1^·cm^−1^ at 260 nm [69]; this value ranged from 2.50 × 10^−4^ to 1.88 × 10^−3^ M in subsequent samples with Co(II)–PicHA–AlaSal complexes. Spectroscopic spectra were run within the range of 200–600 nm [25].

In order to determine the stability of the Co(II)–PicHA–AlaSal system, successive absorption spectra were recorded. The solution containing the Co(II)–PicHA–AlaSal system was prepared in an aqueous 5 mM Tris-HCl/NaCl buffer adjusted to a pH of about 7.2, at PicHA:AlaSal:Co(II) molar ratio 2:2:1 (metal concentration 1.0 × 10^−5^ M). Absorption spectra were recorded within the wavelength range of 200–700 nm over 14 days.

### 3.5. Biological Assays

Biological tests were performed on freshly prepared solutions of PicHA alone, binary complexes in the Co(II)–PicHA system and heteroligand complexes in the Co(II)–PicHA–AlaSal system, as well as those that had been stored for two weeks. The PicHA:AlaSal:Co(II) molar ratio for the heteroligand system was 2:2:1. The compounds were diluted to the following concentrations [mM]: 7.30 (antimicrobial activity tests), 3.65, 1.82, 0.91, 0.46, 0.23, 0.11, 0.06 and 0.03 in the aqueous 5 mM Tris-HCl/NaCl buffer and adjusted to pH 7.2 (NaOH).

#### 3.5.1. Antimicrobial Activity

The reference bacterial strains from the American Type Culture Collection (ATCC, Manassas, VA, USA), including Gram-positive strains *Staphylococcus aureus* ATCC 6538 and ATCC 29213, *Staphylococcus epidermidis* ATCC 12228 and *Enterococcus faecalis* ATCC 2921 as well as Gram-negatives strains ATCC 29212, *Pseudomonas aeruginosa* ATCC 27853, *Helicobacter pylori* ATTC 700392, and three fungal strains: *Candida albicans* ATCC 10231, *Candida glabrata* ATCC 2001, and *Candida parapsilosis* ATCC 22019. One strain of *H. pylori* CCUG 17874 was from the Culture Collection, University of Gothenburg (CCUG), Gothenburg, Sweden.

Antibacterial or antifungal activities were determined by the broth microdilution assay according to European Committee on Antimicrobial Susceptibility (EUCAST) recommendations, as a previously described [25]. The antimicrobial activity of the formulations was evaluated based on their minimal inhibitory concentrations (MIC) and minimal bactericidal concentrations (MBC) or minimal fungicidal concentrations (MFC). MIC was defined as the lowest concentrations resulting in total growth inhibition. The above-mentioned tests were performed in three independent experiments. Amphotericin B, gentamicin and amoxicillin were used as standard antimicrobials.

#### 3.5.2. Cell Cultures

L929 mouse fibroblasts (LGC Standards, Middlesex, UK) were used for in vitro cytotoxicity testing. The cells were maintained under standard conditions of the cell culture incubator (37 °C, 5% CO_2_) in 25-cm^2^ tissue culture flasks in RPMI 1640 medium supplemented with 10% FBS (Biowest, Nuaillé, France) and the following antibiotics: 100 U·mL^−1^ penicillin and 100 µg·mL^−1^ streptomycin, as previously described [25].

##### Measurements of Cellular Metabolic Activity and Global Growth Inhibition

The potential harmful effect of studied samples on L929 cells was assessed by screening the ability of the cells to reduce MTT as recommended by the Food and Drug Administration and ISO norm 109935 to evaluate the morphology of cells [ISO 10993–10995:2009. Biological evaluation of medical devices—Part 5: Tests for in vitro cytotoxicity], as a previously described [25]. Results are presented as the mean percentage ± standard deviation (SD) of the treated cells versus untreated cells (used in triplicates), in four independent experiments. The effectiveness of MTT reduction by treated cells versus untreated cells was calculated based on the following formula:

MTT reduction (%) = (absorbance of treated cells/absorbance of untreated cells × 100%) − 100%.

##### DAPI Staining of Cell Nuclei

The nuclei of L929 cells were stained as previously described [70], using the fluorescent dye DAPI (Sigma-Aldrich, Saint Louis, MO, USA), which has a strong affinity to the AT base pair in DNA. Cell nuclei viewed under a fluorescent microscope (Zeiss, Axio Scope, A1, Jena, Germany), at a wavelength of 358 nm (excitation) and 461 nm (emission). The percentage of cells with blebbing nuclei was assessed.

#### 3.5.3. Statistical Analysis

The results were evaluated using the Kruskal–Wallis test. Statistical significance was accepted at *p*-value < 0.05. Data are presented as mean values ± SD. The statistical analysis was performed using STATISTICA 12 PL software (Stat Soft, Poland).

## 4. Conclusions

Our potentiometry results confirmed the presence of five heteroligand complexes in the equilibrium mixture formed by 2-picolinehydroxamic acid (PicHA) and *N*-(2-hydroxybenzyl)alanine (AlaSal) with Co(II). Despite the presence of the PicHA, allowing coordination with the metal at pH of around 2, heteroligand species formation took place at pH values above 4, as supported by UV–Vis studies. These conditions allow AlaSal to chelate the Co(II) ion following deprotonation of the carboxyl and amino groups. The stability constants of the heteroligand complexes was calculated by potentiometric titrations of the mixed ligand system. A stacking interaction takes place between the AlaSal phenol ring and the PicHA pyridine ring; this increases the stability of the heteroligand complexes compared to their binary species. This is confirmed by more positive values of the$\;\Delta \log_{10}K$ parameter than those predicted for the octahedral geometries.

The absorption spectra indicate that the heteroligand complexes present in an aqueous solution at physiological pH can bind to DNA. The decrease of absorbance at 304–440 nm indicates an intercalative binding resulting from the strong stacking interaction between ligand aromatic chromophores and the DNA base pairs. The heteroligand species present in the equilibrium mixture demonstrated antimicrobial activities against the tested strains: *Staphylococcus aureus*, *Staphylococcus epidermidis* and *Candida parapsilosis*; however, the most effective antimicrobial activity was demonstrated against two *Helicobacter pylori* strains and towards *Candida albicans* and *Candida glabrata* fungi. These results were considered promising because microorganisms such as *H. pylori* and *Candia* co-existing in the gastric mucosa can be both involved in the development of gastric diseases. The tested Co(II)–PicHA–AlaSal complexes within the range 0.03–0.46 mM did not show cytotoxic activity towards L929 cells. However, both the freshly prepared solutions containing complexes, and those stored for two weeks at higher concentrations (0.91–3.65 mM) significantly diminished the metabolic activity of L929 cells. The observation that the stored complexes appeared to have a stronger inhibitory effect on cell growth than the freshly prepared ones may have been due to the heteroligand complexes increasing their concentration over time. Therefore, in order to obtain antimicrobial effects, cytotoxic concentrations are necessary. This limits the application of tested compounds to serve as antimicrobial drugs. However, the fact that the heteroligand complexes demonstrated a greater cytotoxic effect than the ligands and Co(II)–PicHA binary complexes favours the future use of the Co(II)–PicHA–AlaSal complexes in cytotoxic concentrations against cancer cells; further research is needed for this. It is important to note that Co(II)–PicHA–AlaSal may have potential for the treatment of *H. pylori* infections, which may result in the development of gastric cancer [71]. Therefore, performance of MTT assay with cancerous cell lines, including gastric cancer cells is highly recommended. Recently Huang et al. [72], have considered new forms of cobalt oxide nanoparticle based anticancer therapy.

The interaction between the Co(II)–PicHA–AlaSal complexes and DNA likely prevents nuclear replication and ultimately induces eukaryotic cell apoptosis; however, under the experimental conditions used in this study, only a certain percentage of the cells show signs of nuclear degradation. It could be due to another target than DNA. Different intracellular proteins in cancer cells can be a potential targets for cobalt complexes, which may promote anticancer effects due to induction of autophagy or cell cycle arrest, and inhibition of invasion [59].

It seems advisable to continue studies of the antimicrobial activity of individual complexes in an equilibrium mixture at physiological pH. These compounds may constitute the basis for the development of new preparations against drug-resistant strains. Further modification and improvement of the currently tested formulation may allow potentially lower therapeutic concentrations to be used, to eradicate bacteria or inhibition their growth in vivo. It is also worth continuing research to verify the synergistic effects between the tested formulations and currently used antibacterial preparations. Further studies are also necessary to demonstrate the inhibition of neoplastic cell growth by these formulations.

## Figures and Tables

**Table 1:** Decimal logarithms of overall formation constants *β_mll’h_* = [M*_m_*L*_l_*L’*_l’_*H*_h_*]/[M]*^m^*[L]*^l^*[L’]*^l’^*[H]*^h^* at 25.0 ± 0.1 °C, *I* = 0.1. The standard deviations given in parentheses after overall stability constants refer to random errors only.

Species	log_10_ *β_mll’h_*	Related Constants ^b^
[CoL(L’H)] (**1**)	21.40 (4)	10.67 ^c^
[CoLL’]^−^ (**2**)	13.56 (2)	13.56
[CoL_2_ (L’H)]^−^ (**3**)	25.61 (7)	14.88 ^d^
[CoL(L’_2_H)]^2−^ (**4**)	26.75 (5)	16.02 ^e^
[CoLL’_2_]^3−^ (**5**)	16.87 (5)	16.87
*σ*; *n*^a^	12.25; 753	

^a^*σ—*the value of the normalized sum of squared residuals; *n—*number of titration points; ^b^ $\log\beta_{L\prime H}=10.73$ [26]; ^c^ $\log_{10}K_{\mathrm{CoL}(L\prime H)}^{\mathrm{Co}}=\log\beta_{\mathrm{CoL}(L\prime H)}-\log\beta_{L\prime H}$; ^d^ $\log_{10}K_{{\mathrm{CoL}}_{2}\left(L\prime H\right)}^{\mathrm{Co}}=\log\beta_{{\mathrm{CoL}}_{2}\left(L\prime H\right)}-\log\beta_{L\prime H}$; ^e^ $\log_{10}K_{{\mathrm{CoL}(L\prime }_{2}H)}^{\mathrm{Co}}=\log\beta_{{\mathrm{CoL}(L\prime }_{2}H)}-\log\beta_{L\prime H}$.

**Table 2:** Antimicrobial activity of tested compounds, prepared directly and stored for two weeks, shown as minimal inhibitory concentration (MIC) and minimal bactericidal concentration (MBC) or minimal fungicidal concentration (MFC).

Microorganism	MIC/MBC/MFC (mM)		MIC = MBC/MFC (mM)		
	Co(II)–PicHA–AlaSal Complexes		Gentamicin	Amphotericin B	Amoxicillin
	MIC	MBC/MFC			
Gram-negative bacteria					
*Pseudomonas aeruginosa* ATCC 27853	7.30	>7.30	<0.008	-	-
*Escherichia coli* ATCC 25922	7.30	>7.30	<0.004	-	-
*Helicobacter pylori* CCUC 17874	3.65	3.65	-	-	<0.001
*Helicobacter pylori* ATCC 700392	3.65	3.65	-	-	<0.001
Gram-positive bacteria					
*Enterococcus faecalis* ATCC 29212	7.30	>7.30	<0.26	-	-
*Staphylococcus aureus* ATCC 29213	3.65	>7.30	<0.002	-	-
*Staphylococcus aureus* ATCC 6538	3.65	>7.30	<0.002	-	-
*Staphylococcus epidermidis* ATCC 12228	3.65	>7.30	<0.002	-	-
Fungi					
*Candida albicans* ATTC 10231	3.65	3.65	-	<0.001	-
*Candida glabrata* ATCC 2001	3.65	3.65	-	<0.001	-
*Candida parapsilosis* ATCC 22019	3.65	7.30	-	<0.001	-

Gentamicin, amoxicillin and amphotericin B, broad-spectrum antibiotics, used as antibacterial and antifungal reference substances, respectively; (-) not tested.

## Data Availability

Data is contained within the article and Supplementary Materials.

## Supplementary Materials

The following are available online at https://www.mdpi.com/article/10.3390/ph14121254/s1, Figure S1: (a) Spectra of the complexes in the Co(II)–PicHA–AlaSal system at PicHA:AlaSal:Co(II) molar ratio 2:2:1, during titration within the pH range of 1.95–11.18; CCo(II) = 1 × 10−3 M. (b) Extended part of the spectra within the pH range of 1.95–3.87. (c) Molar absorption coefficients (ε) for the complexes in the Co(II)–PicHA–AlaSal system. Figure S2: Spectra of the complexes in the Co(II)–PicHA–AlaSal system in 5 mM Tris-HCl/NaCl buffer at pH 7.2 recorded over 14 days (CCo(II) = 1.0 × 10−5 M, PicHA:AlaSal:Co(II) molar ratio 2:2:1). Figure S3: (a) Species distribution curves as a function of pH relative to PicHA, for the complexes formed in the Co(II)–PicHA–AlaSal system at PicHA:AlaSal:Co(II) molar ratio 2:2:1, CCo(II) = 5.0 × 10−3 M, (b) extended part of species distribution curves. Figure S4: (a) Species distribution curves as a function of pH relative to AlaSal, for the complexes formed in Co(II)–PicHA–AlaSal system at PicHA:AlaSal:Co(II) molar ratio 2:2:1, C_Co(II)_ = 5.0 × 10^−3^ M, (b) extended part of species distribution curves.
