# Supplementary material for: Chemical Characterization and Biological Evaluation of New Cobalt(II) Complexes with Bioactive Ligands, 2-Picolinehydroxamic Acid and Reduced Schiff Base N-(2-Hydroxybenzyl)alanine, in Terms of DNA Binding and Antimicrobial Activity
Source: Pharmaceuticals (Basel). 2021 Dec 2;14(12):1254. doi: 10.3390/ph14121254 (PMC8706952; doi:10.3390/ph14121254)
Supplement: Supplementary file 1 [file pharmaceuticals-14-01254-s001.zip › pharmaceuticals-1478938-supplementary.pdf]

Supplementary Information for the paper

Chemical characterization and biological evaluation of new cobalt(II) complexes with bioactive ligands, 2-picolinehydroxamic acid and reduced Schiff base *N*-(2-hydroxybenzyl)alanine, in terms of DNA binding and antimicrobial activity

Magdalena Woźniczka [1,*], Marta Lichawska [1], Manas Sutradhar [2], Magdalena Chmiela [3], Weronika Gonciarz [3], Marek Pająk [1]

Page 2. **Figure S1. (a)** Spectra of the complexes in the Co(II)–PicHA–AlaSal system at PicHA-to-AlaSal-to-Co(II) molar ratio 2:2:1, during titration within the pH range of 1.95 – 11.18; $C_{Co(II)} = 1 \times 10^{-3}$ M. **(b)** Extended part of the spectra within the pH range of 1.95 – 3.87. **(c)** Molar absorption coefficients (ε) for the complexes in the Co(II)–PicHA–AlaSal system.

Page 3. **Figure S2.** Spectra of the complexes in the Co(II)–PicHA–AlaSal system in 5 mM Tris-HCl/NaCl buffer at pH 7.2 recorded over 14 days ($C_{Co(II)} = 1.0 \times 10^{-5}$ M, PicHA-to-AlaSal-to-Co(II) molar ratio 2:2:1).

Page 4. **Figure S3. (a)** Species distribution curves as a function of pH relative to PicHA, for the complexes formed in the Co(II)–PicHA–AlaSal system at PicHA-to-AlaSal-to-Co(II) molar ratio 2:2:1, $C_{Co(II)} = 5.0 \times 10^{-3}$ M, **(b)** extended part of species distribution curves.

Page 5. **Figure S4. (a)** Species distribution curves as a function of pH relative to AlaSal, for the complexes formed in Co(II)–PicHA–AlaSal system at PicHA-to-AlaSal-to-Co(II) molar ratio 2:2:1, $C_{Co(II)} = 5.0 \times 10^{-3}$ M, **(b)** extended part of species distribution curves.

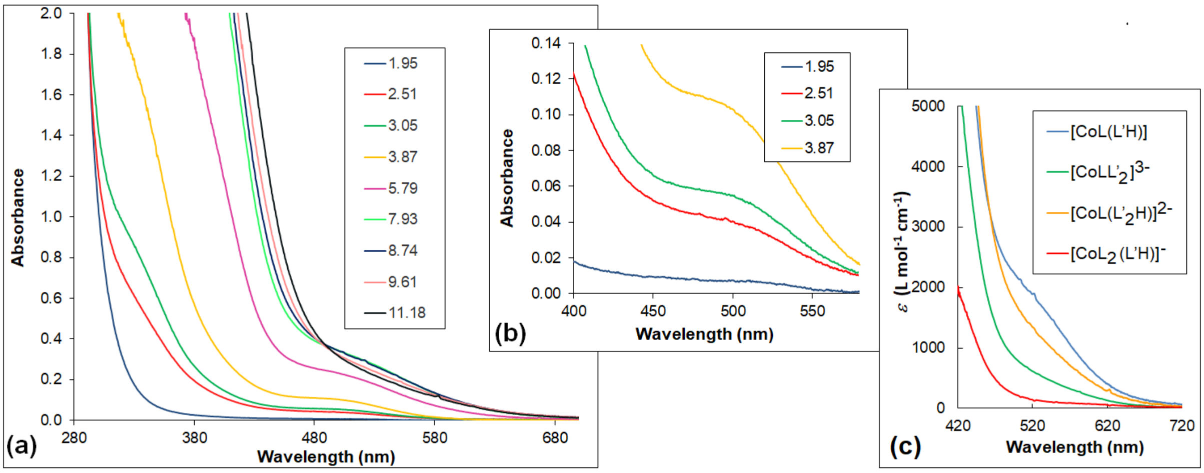

Figure S1. (a) Spectra of the complexes in the Co(II)–PicHA–AlaSal system at PicHA-to-AlaSal-to-Co(II) molar ratio 2:2:1, during titration within the pH range of 1.95 – 11.18; $C_{Co(II)} = 1 \times 10^{-3}$ M. **(b)** Extended part of the spectra within the pH range of 1.95 – 3.87. **(c)** Molar absorption coefficients (ε) for the complexes in the Co(II)–PicHA–AlaSal system.

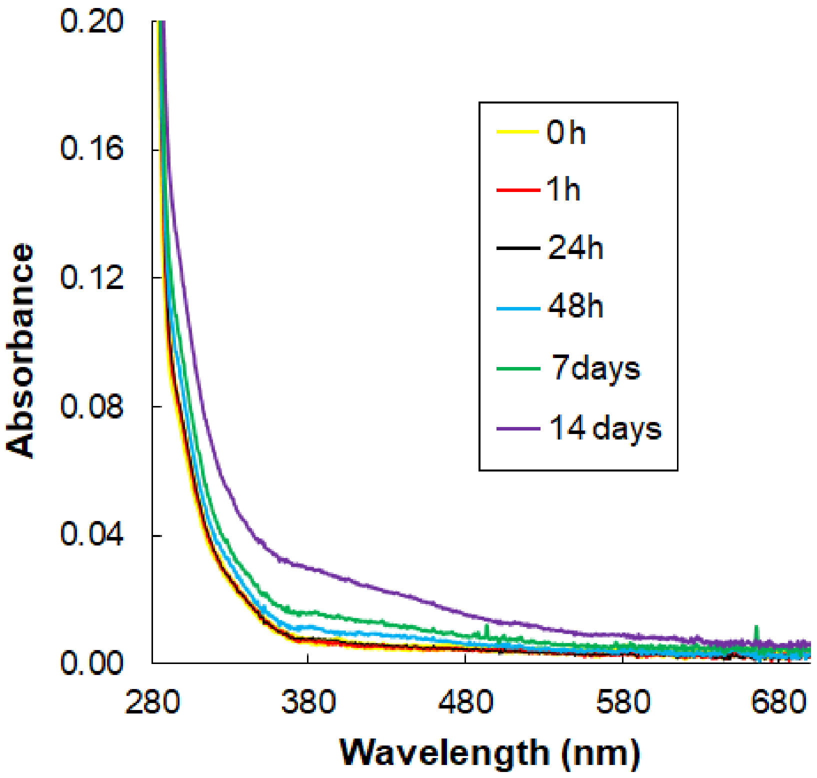

Figure S2. Spectra of the complexes in the Co(II)–PicHA–AlaSal system in 5 mM Tris-HCl/NaCl buffer at pH 7.2 recorded over 14 days ($C_{Co(II)} = 1.0 \times 10^{-5}$ M, PicHA-to-AlaSal-to-Co(II) molar ratio 2:2:1).

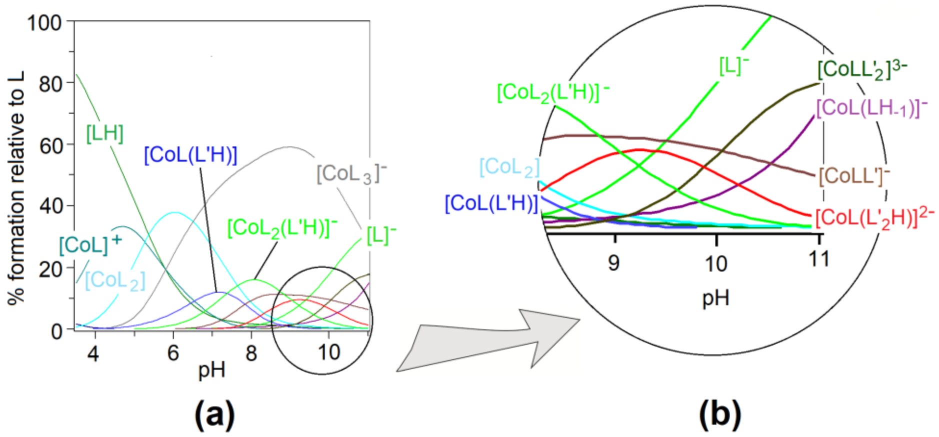

Figure S3. (a) Species distribution curves as a function of pH relative to PicHA, for the complexes formed in the Co(II)–PicHA–AlaSal system at PicHA-to-AlaSal-to-Co(II) molar ratio 2:2:1, $C_{Co(II)} = 5.0 \times 10^{-3}$ M, **(b)** extended part of species distribution curves.

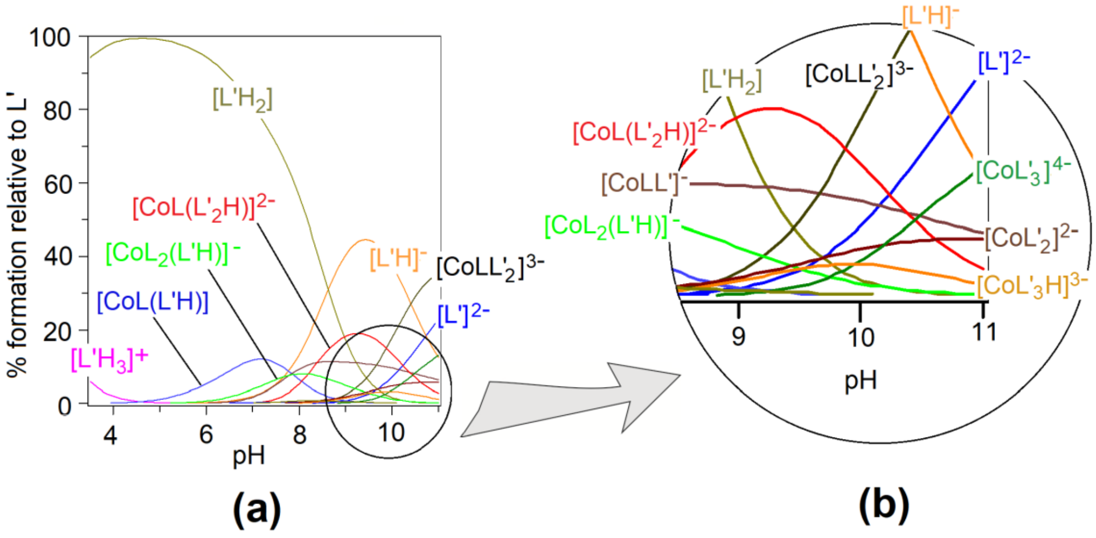

Figure S4. (a) Species distribution curves as a function of pH relative to AlaSal, for the complexes formed in Co(II)–PicHA–AlaSal system at PicHA-to-AlaSal-to-Co(II) molar ratio 2:2:1, $C_{Co(II)} = 5.0 \times 10^{-3}$ M, **(b)** extended part of species distribution curves.
